# Fixed-dose combination antihypertensive medications, adherence, and clinical outcomes: A population-based retrospective cohort study

**DOI:** 10.1371/journal.pmed.1002584

**Published:** 2018-06-11

**Authors:** Amol A. Verma, Wayne Khuu, Mina Tadrous, Tara Gomes, Muhammad M. Mamdani

**Affiliations:** 1 Li Ka Shing Centre for Healthcare Analytics Research and Training, St. Michael’s Hospital, Toronto, Ontario, Canada; 2 Eliot Phillipson Clinician-Scientist Training Program, Department of Medicine, University of Toronto, Toronto, Ontario, Canada; 3 Institute for Clinical and Evaluative Sciences, Toronto, Ontario, Canada; 4 Leslie Dan Faculty of Pharmacy, University of Toronto, Toronto, Ontario, Canada; 5 Institute of Health Policy, Management, and Evaluation, University of Toronto, Toronto, Ontario, Canada; The George Institute for Global Health, AUSTRALIA

## Abstract

**Background:**

The majority of people with hypertension require more than one medication to achieve blood pressure control. Many patients are prescribed multipill antihypertensive regimens rather than single-pill fixed-dose combination (FDC) treatment. Although FDC use may improve medication adherence, the impact on patient outcomes is unclear. We compared clinical outcomes and medication adherence with FDC therapy versus multipill combination therapy in a real-world setting using linked clinical and administrative databases.

**Methods and findings:**

We conducted a population-based retrospective cohort study of 13,350 individuals 66 years and older in Ontario, Canada with up to 5 years of follow-up. We included individuals who were newly initiated on one angiotensin-converting enzyme inhibitor (ACEI) or angiotensin II-receptor blocker (ARB) plus one thiazide diuretic. High-dimensional propensity score matching was used to compare individuals receiving FDC versus multipill therapy. The primary outcome was a composite of death or hospitalization for acute myocardial infarction (AMI), heart failure, or stroke. We conducted 2 analyses to examine the association between adherence and patient outcomes. First, we performed an on-treatment analysis to determine whether outcomes differed between groups while patients were on treatment, censoring patients when they first discontinued treatment, defined as not receiving medications within 150% of the previous days’ supply. Second, we conducted an intention-to-treat analysis that followed individuals allowing for breaks in treatment to quantify the difference in drug adherence between groups and assess its impact on clinical outcomes. As expected, there was no significant difference in the primary outcome between groups in the on-treatment analysis (HR 1.06, 95% CI 0.86–1.31, *P* = 0.60). In the intention-to-treat analysis, the proportion of total follow-up days covered with medications was significantly greater in the FDC group (70%; IQR 19–98) than in the multipill group (42%, IQR 11–91, *P* < 0.01), and the primary outcome was less frequent in FDC recipients (3.4 versus 3.9 events per 100 person-years; HR 0.89, 95% CI 0.81–0.97, *P* < 0.01). The main limitations of this study were the lack of data regarding cause of death and blood pressure measurements and the possibility of residual confounding.

**Conclusions:**

Among older adults initiating combination antihypertensive treatment, FDC therapy was associated with a significantly lower risk of composite clinical outcomes, which may be related to better medication adherence.

## Abstract 2

In a retrospective cohort study using clinical data from Ontario, Canda, Amol Verma and colleagues compare medication adherence and clinical outcomes among people receiving single-pill versus multiple pill regimens of blood pressure treatment.

## Author summary

**Why was this study done?:**

- Most people with hypertension require more than one medication to achieve blood pressure control.
- Multiple medications can be combined into a single pill or prescribed as separate pills.
- Patients are more likely to adhere to single-pill rather than multiple-pill treatment regimens.
- It is unclear whether clinical outcomes are better in patients who receive single-pill combinations or multiple separate pills and whether this might be related to medication adherence.

**What did the researchers do and find?:**

- We used administrative and clinical databases in Ontario, Canada to compare adults 65 years and older who were starting combination blood pressure treatment with either single-pill or multiple-pill regimens.
- We used advanced statistical methods to identify a cohort of individuals who were comparable.
- We found that people who received single-pill combinations had a significantly lower rate of the combined outcome of death or hospitalization for heart attack, heart failure, or stroke and that these differences were related to better medication adherence.

**What do these findings mean?:**

- Approximately 675 million people globally require combination antihypertensive therapy, and up to 40% of patients with hypertension in high-income countries are treated with multiple-pill regimens.
- Our study suggests that single-pill combination treatment is associated with markedly improved medication adherence and better clinical outcomes.
- Using single-pill combinations rather than multiple pills may represent a simple and potentially low-cost intervention that could substantially reduce the global burden of death and disability related to hypertension.

## Introduction

Hypertension affects an estimated 900 million adults [1] and is the leading cause of global death or disability [2]. Approximately 75% of people with hypertension require more than one medication to achieve blood pressure control [3]. Although many hypertension management guidelines recommend initiating combination treatment with either separate drugs in multipill combinations or single-pill fixed-dose combinations (FDCs) [4–6], only half of national hypertension societies recommend FDC treatment [7], which may be due to a lack of evidence about effect on clinical outcomes. Both FDC and multipill regimens are common in clinical practice. Studies in high-income countries report that 22% to 43% of patients receive multipill regimens when initiated on combination antihypertensive therapy [8,9].

The blood pressure–lowering effect of FDC therapy was similar to multipill regimens in a meta-analysis of 9 trials [10], which were all less than 1 year in duration. However, blood pressure control was found to be worse with FDC over 6 years’ follow-up in the Swiss Hypertension Cohort Study [11]. It may not be possible to extrapolate the effectiveness of FDC therapy for hypertension from clinical trials to real-world settings. Although FDC use has been associated with improved medication adherence compared with multipill therapy in both clinical trials and observational settings [8,10,12,13], critics of FDC therapy argue that it makes dose titration or changing medications more difficult and that this could lead to poorer outcomes [14,15]. It is not known whether improved adherence related to FDC therapy translates into better clinical outcomes.

We examined the association between initiating FDC versus multipill antihypertensive therapy and cardiovascular events or death in a real-world setting. Addressing this question in a real-world setting is particularly important because the differences between FDC and multipill therapy arise from the way medications are used, and patterns of medication use in clinical trials may not be generalizable [16].

## Methods

### Setting and design

We conducted a population-based, propensity score-matched, retrospective cohort study of residents of Ontario, Canada aged 66 years or older who initiated combination antihypertensive therapy between April 1, 2004 and December 31, 2014. Individuals were followed for 5 years or until March 31, 2015. Provincial health insurance in Ontario covers physician and hospital services for all residents and prescription drugs for those over 65 years of age. This study was conducted using a prespecified analysis plan approved by the Sunnybrook Health Sciences Centre Research Ethics Board. This study is reported as per the RECORD guidelines (S1 Checklist).

### Sources of data

We used the Ontario Drug Benefit claims database, which records prescription medications dispensed to all Ontarians over the age of 65, to determine exposure to combination antihypertensive therapy. Data pertaining to hospitalizations and emergency department use were obtained from the Canadian Institute for Health Information Discharge Abstract Database and National Ambulatory Care Reporting System. The Registered Persons Database was used to obtain basic demographic information and date of death for all Ontario residents. Demographic and specialty data for all physicians practicing in Ontario were obtained from the Institute for Clinical Evaluative Sciences (ICES) Physicians Database, and the Ontario Health Insurance Plan claims database was used to identify claims for all insured physician services. These databases have excellent data completeness and quality [17], and they were anonymously linked using encrypted person-level identifiers, as in previous studies [18–20].

### Cohort design

We identified a cohort of new users of combination antihypertensive medication who were prescribed one angiotensin-converting enzyme inhibitor (ACEI) or angiotensin II-receptor blocker (ARB) plus one thiazide diuretic, either as FDC or as a multipill combination. These medication combinations were selected because they are common guideline-recommended options for initial antihypertensive therapy [4,5] and to avoid potential confounding when comparing combinations of various medication classes. We included only new users of antihypertensive medications to avoid selection bias based on prior medication adherence. New users were defined as receiving no prescription for any antihypertensive medication in the year prior to study enrollment.

The index date for study enrollment was defined as the date of first prescription of antihypertensive medications. To match FDC therapy, in which both medications are taken together, the multipill combination group included only individuals who were dispensed both medications on the same index date. Initiating multipill combination therapy on separate days might reflect treatment intensification in response to failure of monotherapy and select for a higher-risk population, therefore these individuals were not included.

**Fig 1:**
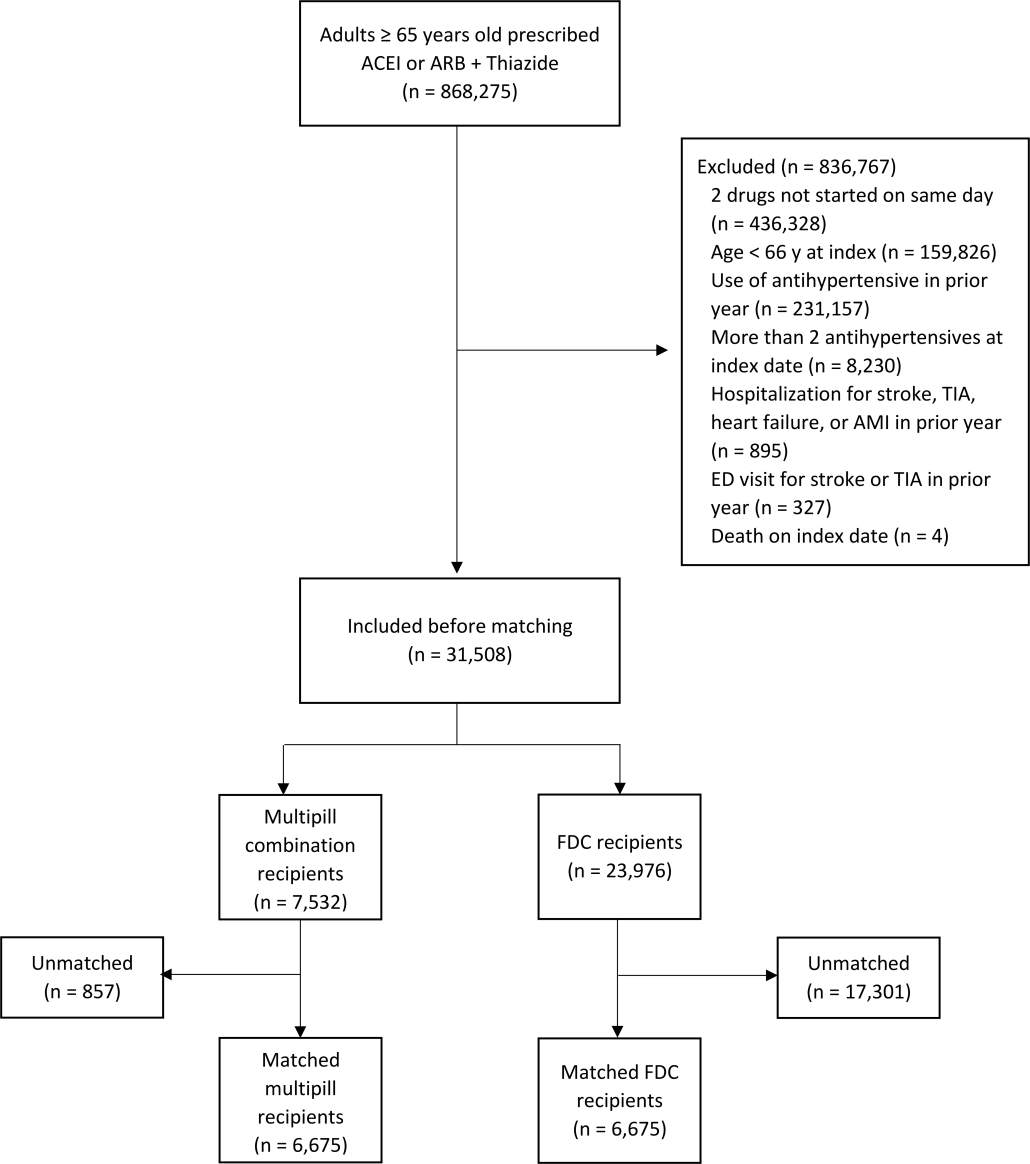
Cohort creation diagram. ACEI, angiotensin-converting enzyme inhibitor; AMI, acute myocardial infarction; ARB, angiotensin II-receptor blocker; ED, emergency department; FDC, single-pill fixed-dose combination; TIA, transient ischemic attack.

We excluded individuals with any hospitalization for stroke, transient ischemic attack (TIA), heart failure, or myocardial infarction in the year prior to study enrollment to reduce selection bias that might arise from differential prescribing of FDC versus multipill combinations after hospitalization. We also excluded individuals with any emergency department visit for stroke or TIA in the year prior to study enrollment because the combination of ACEI and thiazide may be used for secondary stroke prevention even among nonhypertensive adults [21]. Finally, we excluded individuals who were prescribed any antihypertensive medications in addition to the initial combination therapy on the day of study enrollment. See cohort flow diagram for details (Fig 1).

To minimize selection bias, we used high-dimensional propensity score matching to identify comparable groups [22]. Each multipill combination user was matched with one FDC user based on the dose of index antihypertensive medications and the propensity score (allowing no more than a difference of 20% of the SD of the logit of the propensity score between matched pairs). The following datasets were used to develop the high-dimensional propensity score based on data from the year prior to study enrollment: prescription drug claims, diagnosis codes, and procedure codes from all hospitalizations and emergency department visits as well as insurance claims and diagnosis codes for physician services. In total, this represented 7 dimensions of data, and the 200 most prevalent codes from each dimension were retained as candidate covariates. All potential covariates were sorted in descending order by the magnitude of the log of the multiplicative bias term as previously described [22], and the top 500 covariates were included in the propensity score model. We also included the following prespecified covariates: age, sex, income quintile, year of the index date to account for possible changes in clinical practice over the duration of the study period, Charlson comorbidity index, the number of outpatient physician visits in the year prior to study enrollment, and any cardiology visit in the 3 months prior to study enrollment. The high-dimensional propensity score was calculated using version 2.4.4 of the freely available macro developed at Harvard University [23].

The dose of index antihypertensive medications was used as a proxy for severity of hypertension at baseline. As described in the supporting information, each medication was categorized into high- and low-dose categories based on the usual daily dose range described in clinical practice guidelines (S1 Table) [24]. Individuals were then categorized into 3 groups based on the dose of their index antihypertensives: “low” if both medications were low-dose, “high” if both medications were high-dose, and “intermediate” if only one medication was high-dose.

We conducted 2 complementary analyses to disentangle whether differences in outcomes between FDC and multipill regimens might be related to improved adherence or rather, differences in the actual effects of medications between groups. First, we performed an on-treatment analysis, censoring patients when they first discontinued treatment. Therefore, outcomes were assessed only during active treatment, which removed the effect of adherence. Second, we conducted an intention-to-treat analysis that followed individuals irrespective of disruptions in treatment to quantify the difference in drug adherence between groups and assess its impact on clinical outcomes.

We present 2 measures to describe adherence to antihypertensive medications based on medication dispensing: the time to the first instance of discontinuation and the proportion of total days covered. Discontinuation of antihypertensive medication was defined as no repeat prescription within 150% of the previous days’ supply. For example, a medication would be considered discontinued if the index prescription was dispensed for 30 days and a second prescription was not dispensed within 45 days. For the multipill combination group, repeat prescription of both medications within the 150% grace period was necessary to be considered continuous use. Medication class switching between ACEI or ARB or between different thiazides was not considered discontinuation. The on-treatment analysis followed individuals until the first instance of discontinuation. Because individuals may subsequently receive the index antihypertensive medications after an initial disruption in therapy, we also calculated the total duration of use of the index antihypertensive medications during follow-up. This was reported as the proportion of days of follow-up covered by the index antihypertensive medications in all periods of continuous use.

We hypothesized that, if the benefit of FDC therapy was related to improved medication adherence, the intention-to-treat analysis would demonstrate significant between-group differences in clinical outcomes whereas the on-treatment analysis would not.

### Outcomes

The primary outcome was a composite of all-cause death and hospitalization for acute myocardial infarction (AMI), heart failure, or stroke. The administrative diagnostic codes (S6 Table) used to define the primary outcome have good sensitivity and positive predictive value, respectively: AMI, 89% and 87%; heart failure, 79% and 85%; and stroke, 76% and 97% [25,26]. Secondary outcomes included each individual component of the primary outcome, hospitalization for hypokalemia or hyponatremia to assess for safety, and the first instance of discontinuation of antihypertensive medication. Because we expected no association between antihypertensive treatment and cataract surgery, this was used as a “tracer outcome” to assess for residual confounding between the groups.

### Statistical analysis

Propensity score matching resulted in balanced groups with no baseline covariates differing by more than 0.1 standardized difference except for index medication, which differed because ARBs were more common in FDC formulations than ACEI. Adjusting for covariates with less than 0.1 standardized difference imbalance provides negligible benefits in addressing residual confounding [27]. Thus, no additional covariates were included in the regression models. In the on-treatment analysis, individuals were censored when they first discontinued antihypertensive medications. The median time to the first instance of medication discontinuation was compared between the FDC and multipill combination groups using Kaplan-Meier estimators and the log-rank test. The proportion of days covered by antihypertensive medications in both groups was compared using the Wilcoxon signed rank sum test. Our primary analysis was a time-to-event analysis in the matched cohort using Cox proportional hazards regression including a robust variance estimator that accounted for clustering within matched sets [28]. When analyzing each component of the primary composite outcome separately, individuals were also censored for death. Proportional hazards assumptions were tested for the primary outcome using a time-dependent covariate by including an interaction term between the antihypertensive group and time in the Cox models. All of the above analyses were prespecified, with the exception of the calculation of the proportion of days covered by index antihypertensive medications, which was a post hoc analysis conducted to better describe medication use after we recognized that many participants continued to receive index medications after an instance of medication discontinuation.

The following additional analyses were provided in response to peer review comments. First, we conducted a sensitivity analysis by defining medication discontinuation as no repeat prescription within 300% of the previous days’ supply. Second, to better understand the role that concomitant cardiovascular risk-modifying treatments may have played in influencing our findings, we described the receipt of other cardiovascular medications in the last 90 days of follow-up in the FDC and multipill groups. A standardized difference less than 0.1 between groups was considered to be well-balanced. Finally, to better understand patterns of medication use, we reported antihypertensive and other cardiovascular medications dispensed in the last 90 days of follow-up among individuals who did and did not discontinue their index antihypertensive medications, defined as having any break in therapy of greater than 150% of the previous days’ supply.

All analyses were performed using SAS software (version 9.4).

## Results

### Study cohort and follow-up

After propensity score matching, we identified a cohort of 13,350 individuals (6,675 in each group) who were new users of combination antihypertensive therapy with an ACEI or ARB plus a thiazide diuretic. In the intention-to-treat analysis, median follow-up time was 1,826 days in both the FDC group (interquartile range 1,163–1,826) and the multipill group (IQR 1,142–1,826).

### Baseline characteristics

The 2 groups were well-balanced on all baseline characteristics except medication class at index (Table 1) and were relatively similar before matching (S3 Table). The median age at index was 71 years (IQR 68–77). In both groups, 42.7% of individuals received low-dose medication, 43.0% received intermediate-dose, and 14.3% received high-dose. FDC users were more likely to receive an ARB (65.1% versus 23.3% in multipill group) than an ACEI and more likely to receive hydrochlorothiazide (88.2% versus 82.9% in multipill group) than other thiazides.

**Table 1:** Baseline characteristics in propensity score–matched study cohort.

Characteristic	Multipill	FDC	Standardized Difference
	(*N* = 6,675)	(*N* = 6,675)	
**Age, median (IQR), y**	71 (68–77)	71 (68–77)	0
**Female, *n* (%)**	3,680 (55.1)	3,590 (53.8)	0.03
**Neighborhood income quintile, *n* (%)**			
1	1,345 (20.1)	1,403 (21.0)	0.02
2	1,388 (20.8)	1,371 (20.5)	0.01
3	1,303 (19.5)	1,268 (19.0)	0.01
4	1,244 (18.6)	1,231 (18.4)	0.01
5	1,342 (20.1)	1,350 (20.2)	0
Missing	53 (0.8)	52 (0.8)	0
**Nursing home residence, *n* (%)**	121 (1.8)	50 (0.7)	0.09
**Rural residence, *n* (%)**	951 (14.2)	785 (11.8)	0.07
**Charlson comorbidity score, categorized**			
No hospitalizations	5,917 (88.6)	5,923 (88.7)	0
0	471 (7.1)	439 (6.6)	0.02
1	136 (2.0)	163 (2.4)	0.03
2+	151 (2.3)	150 (2.2)	0
**Healthcare utilization**			
Hospitalizations in prior year, mean (SD)	0.09 (0.33)	0.09 (0.34)	0.01
Outpatient physician visits in prior year, median (IQR)	4 (2–8)	4 (2–8)	0
Visit to cardiologist in prior 3 months, *n* (%)	1,148 (17.2)	1,108 (16.6)	0.02
Cardiac catheterization in prior 5 years, *n* (%)	74 (1.1)	57 (0.9)	0.03
Total number of different prescription drugs in prior 100 days, mean (SD)	1.47 (2.17)	1.48 (2.13)	0
**Medical comorbidities, *n* (%)**			
Diabetes^†^	1,129 (16.9)	1,175 (17.6)	0.02
Stroke^††^	45 (0.7)	28 (0.4)	0.03
AMI^††^	17 (0.3)	13 (0.2)	0.01
Heart Failure^††^	40 (0.6)	31 (0.5)	0.02
Peripheral vascular disease^††^	32 (0.5)	33 (0.5)	0
Chronic kidney disease^††^	18 (0.3)	16 (0.2)	0.01
Cancer^†^	673 (10.1)	675 (10.1)	0
Chronic obstructive pulmonary disease^†^	352 (5.3)	348 (5.2)	0
Dementia^††^	349 (5.2)	320 (4.8)	0.02
**Index medication use, *n* (%)**			
ACEI	5,117 (76.7)	2,330 (34.9)	0.93
ARB	1,558 (23.3)	4,345 (65.1)	0.93
Hydrochlorothiazide	5,531 (82.9)	5,887 (88.2)	0.15
Chlorthalidone	73 (1.1)	0 (0.0)	0.15
Indapamide	1,071 (16.0)	788 (11.8)	0.12
**Index medication dose category, *n* (%)**			
Low	2,849 (42.7)	2,849 (42.7)	0
Medium	2,873 (43.0)	2,873 (43.0)	0
High	953 (14.3)	953 (14.3)	0
**Other medications in prior 100 days, *n* (%)**			
Noninsulin antihyperglycemic	673 (10.1)	698 (10.5)	0.01
Insulin	79 (1.2)	97 (1.5)	0.02
Statin	1,693 (25.4)	1,579 (23.7)	0.04
Warfarin	122 (1.8)	98 (1.5)	0.03
Direct oral anticoagulants	8 (0.1)	10 (0.1)	0.01
Digoxin	41 (0.6)	35 (0.5)	0.01
Clopidogrel	70 (1.0)	59 (0.9)	0.02

^†^Diagnosis occurred at any point in time.

^††^Diagnosis occurred within 5 years of cohort entry. Index medication dose categorization is described in S1 Table.

Abbreviations: ACEI, angiotensin-converting enzyme inhibitor; AMI, acute myocardial infarction; ARB, angiotensin II-receptor blocker; FDC, single-pill fixed-dose combination; IQR, interquartile range; SD, standard deviation.

### Medication use

The median time to the first instance of medication discontinuation was 191 days (IQR 45–741) in the FDC group and 150 days (IQR 45–446) in the multipill group (*P* < 0.01, Table 2). Medication discontinuation, defined as any break in therapy of greater than 150% of the previous days’ supply, occurred in 88.7% of individuals receiving multipill treatment and 83.1% in the FDC group (HR 0.80, 95% CI 0.77–0.83, *P* < 0.01). Individuals often resumed use of their index medications after a period of discontinuation (S4 Table). When examining use of the index antihypertensives over the entire study period, the proportion of days covered was 70% in the FDC group (IQR 19%–98%), which was significantly greater than 42% in the multipill group (IQR 11%–91%, *P* < 0.01). The proportion of days covered was similar in the sensitivity analysis using the less stringent definition of medication discontinuation (S5 Table).

**Table 2:** Medication use among individuals treated with multipill or FDC antihypertensive regimens.

Medication Use	Multipill	FDC
	*N* = 6,675	*N* = 6,675
**Follow-up time, median (IQR), days**	1,826 (1,142–1,826)	1,826 (1,163–1,826)
Time to first instance of discontinuation, median (IQR), days*	150 (45–446)	191 (45–741)^†^
**Proportion of total days covered, median (IQR)**	0.42 (0.11–0.91)	0.70 (0.19–0.98)^†^

*Indicates Kaplan-Meier estimate.

^†^Indicates *P* < 0.01 for between-group difference.

Time to first instance of discontinuation represents the first period of continuous medication use, defined as no disruption of greater than 150% of the previous days’ supply in receiving the index medications.

Abbreviations: FDC, single-pill fixed-dose combination; IQR, interquartile range.

Use of other cardiovascular risk-lowering medications was well-balanced between the FDC and multipill groups at baseline (Table 1) and in the last 90 days of follow-up (Table 3). Individuals in the FDC group were more likely to receive their index antihypertensive in the last 90 days of follow-up, but use of other antihypertensive medications was well-balanced (Table 3).

**Table 3:** Use of antihypertensive and cardiovascular medications in the last 90 days of follow-up.

Medication Class	Multipill	FDC	Standardized Difference
	*N* = 6,675	*N* = 6,675	
	*N* (%)	*N* (%)	
**Index antihypertensive**	1,997 (29.9)	2,756 (41.3)	0.24
**Other antihypertensive**	1,925 (28.8)	1,908 (28.6)	0.01
**Noninsulin antihyperglycemic**	813 (12.2)	890 (13.3)	0.03
**Insulin**	149 (2.2)	170 (2.5)	0.02
**Statin**	2,209 (33.1)	2,148 (32.2)	0.02
**Warfarin**	231 (3.5)	201 (3.1)	0.02
**Direct oral anticoagulants**	86 (1.3)	85 (1.3)	0.00
**Digoxin**	NA*	NA*	<0.1*
**Clopidogrel**	215 (3.2)	201 (3.0)	0.01

*Data suppressed to comply with ICES privacy policies because calculations required the use of a cell involving 5 or fewer events.

This table reports the number and proportion of individuals who received each medication class in the last 90 days of follow-up. Standardized difference less than 0.1 was considered well-balanced.

Abbreviations: FDC, single-pill fixed-dose combination; ICES, Institute for Clinical Evaluative Sciences.

### Clinical outcomes in the on-treatment analysis

In the on-treatment analysis following individuals until the first instance of medication discontinuation, there were no significant differences in primary or secondary outcomes between groups (Table 4). The composite primary outcome occurred at a rate of 2.4 events per 100 person-years in both the FDC and multipill groups (HR 1.06, 95% CI 0.86–1.31, *P* = 0.60).

**Table 4:** Clinical outcomes among individuals treated with multipill or FDC antihypertensive regimens, on-treatment analysis.

Outcome	Multipill	FDC	HR^†^ (95% CI)	*P* value
	*N* = 6,675	*N* = 6,675		
	Event Rate* (Events/Years of Follow-up)	Event Rate* (Events/Years of Follow-up)		
**Primary Outcome**	2.4 (149/6,306)	2.4 (198/8,227)	1.06 (0.86–1.31)	0.60
**Secondary Outcomes**				
AMI	0.5 (34/6,322)	0.6 (46/8,258)	1.07 (0.69–1.68)	0.77
Heart failure	0.2 (11/6,330)	0.2 (19/8,261)	1.37 (0.66–2.99)	0.41
Stroke	0.4 (26/6,320)	0.5 (39/8,243)	1.26 (0.77–2.1)	0.37
Death	1.4 (86/6,333)	1.3 (108/8,267)	0.99 (0.75–1.32)	0.94
**Safety Outcomes**				
Hypokalemia	N/A^††^	N/A^††^	N/A^††^	N/A^††^
Hyponatremia	0.2 (11/6,332)	0.2 (14/8,265)	1.10 (0.50–2.49)	0.80
**Tracer Outcome**				
Cataract surgery	5.6 (331/5,946)	5.2 (397/7,663)	0.99 (0.85–1.14)	0.83

The primary outcome was a composite of death or hospitalization with AMI, heart failure, or stroke.

*Event rate per 100 person-years.

^†^HR was calculated with multipill group as the reference category.

^††^Because there were fewer than 5 events, the data were suppressed to comply with ICES privacy policies, and a regression model was not fit.

Abbreviations: AMI, acute myocardial infarction; FDC, single-pill fixed-dose combination; HR, hazard ratio; ICES, Institute for Clinical Evaluative Sciences.

There were also no significant differences between the groups with respect to hospitalizations for hypokalemia or hyponatremia, which occurred in fewer than 0.5% of cases (Table 2). There was no significant difference in the tracer outcome of cataract surgery between the groups (HR 0.99, 95% CI 0.85–1.14, *P* = 0.83).

In a sensitivity analysis using the less stringent definition of medication discontinuation, the results were similar, with the primary outcome occurring at a rate of 2.2 events and 2.1 events per 100 person-years in the FDC group and the multipill group, respectively (HR 1.09, 95% CI 0.92–1.30, *P* = 0.34).

### Clinical outcomes in the intention-to-treat analysis

**Fig 2:**
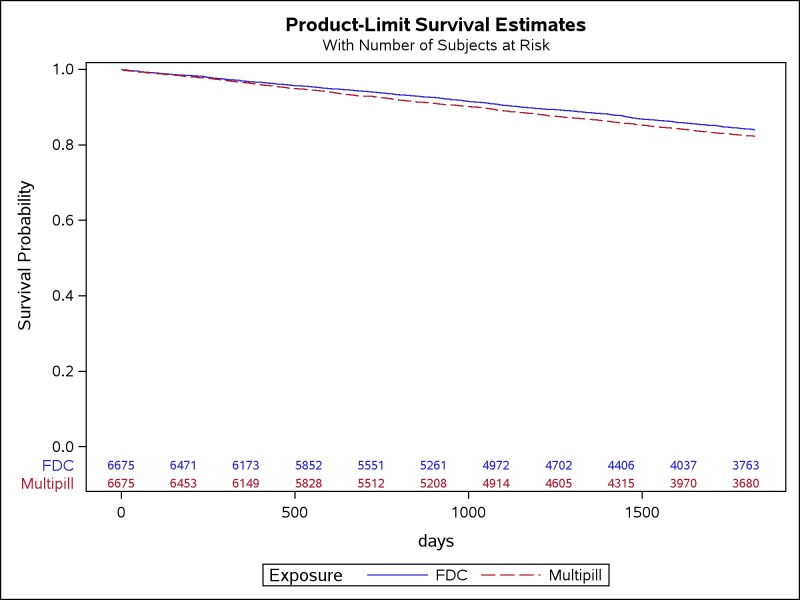
Survival estimates among individuals initiated on FDC versus multipill combination therapy. Legend: Kaplan-Meier estimates of survival probability. FDC, single-pill fixed-dose combination.

The composite primary outcome in the intention-to-treat analysis occurred at a significantly lower rate in the FDC group than the multipill group (3.4 versus 3.9 events per 100 person-years; HR 0.89, 95% CI 0.81–0.97, *P* < 0.01; Table 5, Fig 2). In our analysis of secondary endpoints, the hazard of death was significantly lower among individuals in the FDC compared to the multipill group—2.4 versus 2.8 deaths per 100 person-years, respectively (HR 0.85, 95% CI 0.77–0.94, *P* < 0.01). No significant differences were observed in the hazards for other components of the composite endpoint (Table 5).

**Table 5:** Clinical outcomes among individuals treated with multipill or FDC antihypertensive regimens, primary intention-to-treat analysis.

Outcome	Multipill	FDC	HR^†^ (95% CI)	*P* value
	*N* = 6,675	*N* = 6,675		
	Event Rate* (Events/Years of Follow-up)	Event Rate* (Events/Years of Follow-up)		
**Primary Outcome**	3.9 (1,008/25,967)	3.4 (904/26,226)	0.89 (0.81–0.97)	<0.01
**Secondary Outcomes**				
AMI	0.6 (158/26,376)	0.5 (142/26,569)	0.89 (0.71–1.12)	0.33
Heart failure	0.4 (97/26,526)	0.3 (91/26,605)	0.93 (0.70–1.24)	0.62
Stroke	0.5 (139/26,440)	0.6 (151/26,604)	1.08 (0.86–1.36)	0.51
Death	2.8 (755/26,699)	2.4 (646/26,854)	0.85 (0.77–0.94)	<0.01
Instance of drug discontinuation	93.4 (5,921/6,333)	67.0 (554/8,268)	0.80 (0.77–0.83)	<0.01
**Safety Outcomes**				
Hypokalemia	N/A^**††**^	N/A^**††**^	N/A^**††**^	N/A^**††**^
Hyponatremia	0.1 (35/26,626)	0.1 (30/26,790)	0.85 (0.52–1.39)	0.52
**Tracer Outcome**				
Cataract surgery	4.5 (1,072/24,027)	4.5 (1,089/24,118)	1.01 (0.93–1.10)	0.78

The primary outcome was a composite of death or hospitalization with AMI, heart failure, or stroke.

*Event rate per 100 person-years. An instance of drug discontinuation was defined as receiving no repeat medication within 150% of the previous days’ supply of the index medications.

^†^HR was calculated with multipill group as the reference category.

^††^Because there were fewer than 5 events, the data were suppressed to comply with ICES privacy policies, and a regression model was not fit.

Abbreviations: AMI, acute myocardial infarction; FDC, single-pill fixed-dose combination; HR, hazard ratio; ICES, Institute for Clinical Evaluative Sciences.

There were no significant differences between the groups with respect to hospitalizations for hypokalemia or hyponatremia, which occurred rarely (Table 5). There was no significant difference in the tracer outcome of cataract surgery between the groups (HR 1.01, 95% CI 0.93–1.10, *P* = 0.78).

## Discussion

Among older adults initiating antihypertensive therapy, FDC treatment was associated with a significantly lower risk of composite clinical outcomes compared with multipill treatment, which may be related to better medication adherence. In the on-treatment analysis, outcomes were similar among adults who were actively receiving treatment. However, the intention-to-treat analysis revealed meaningful differences between groups with respect to adherence, with 70% of total days covered during follow-up in the FDC group compared with 42% in the multipill group. This was associated with a significantly lower risk of composite clinical outcomes among the FDC treatment group, with an absolute difference of 0.5 fewer primary outcome events and 0.4 fewer deaths per 100 person-years of follow-up.

We used a novel application of a paired comparison between an intention-to-treat and on-treatment analysis to investigate whether adherence to treatment was related to better clinical outcomes. The on-treatment analysis only compared outcomes in patients up until the first instance of medication discontinuation and revealed that clinical outcomes were not significantly different when there were no medication adherence differences between groups. This contrasted with the intention-to-treat analysis, which followed patients despite disruptions in medication use and in which FDC therapy was associated with better medication adherence and subsequent clinical outcomes. This observation supports the hypothesis that improved medication adherence associated with FDC use confers important clinical benefits in a real-world setting.

The results of several sensitivity analyses support our findings. First, when the definition of medication discontinuation was made less stringent (permitting a 300% grace period instead of 150%), we observed similar results with respect to clinical outcomes and proportion of total days covered by index medications. This suggests that our findings are unlikely to have been biased by our chosen definition of medication discontinuation. Second, the use of other cardiovascular risk-modifying medications was well-balanced between the FDC and multipill groups, both at baseline and in the last 90 days of follow-up. This suggests that our results are unlikely to have been biased by between-group differences in the use of other concomitant therapies.

Blood pressure control often requires multiple medications. More medications and more complex regimens reduce medication adherence [29], whereas simplifying regimens improves adherence [30]. FDC therapy offers an appealing solution by allowing more intensive treatment with a simpler regimen. Gupta and colleagues performed a systematic review and meta-analysis of randomized controlled trials and cohort studies of FDC compared with multipill therapy [10]. They identified 5 studies involving 17,999 individuals and found that FDC was associated with improved adherence (odds ratio 1.21, 95% CI 1.03–1.43). These findings are consistent with another meta-analysis, which included studies of hypertension and other conditions like HIV and which found that FDC therapy was associated with a 26% improvement in medication adherence [12]. A recent large United States study using claims data found that patients initiating FDC for hypertension were 13% more likely to be adherent to medications [8]. Our findings—that FDC use was associated with 28% more days covered with medications and an HR of 0.80 for the first instance of medication discontinuation (95% CI 0.77–0.83)—are very similar to the existing literature, which strengthens our confidence in the generalizability of our results. It is worth noting that provincial health insurance covered the cost of the medications in the study population, and therefore our findings may not be generalizable to settings in which there are substantial out-of-pocket cost differences between FDC and multipill regimens.

Randomized trials and observational studies have not rigorously examined whether FDC use is associated with better clinical outcomes. In an individual-patient data meta-analysis of 3 pragmatic trials, FDC therapy combining antihypertensive and other cardiovascular medications was associated with reduced blood pressure compared to usual care [31]. In their meta-analysis, Gupta and colleagues found a nonsignificant improvement in blood pressure control with FDC therapy [10], but clinical outcomes were not assessed in the included studies. Real-world evidence is contradictory and may reflect unmeasured bias [11,32]. One retrospective cohort study in the United Kingdom found fewer cardiovascular events among patients receiving FDC compared with multipill combinations (HR 0.74, 95% CI 0.70–0.77) [33]. However, this analysis was limited by substantial risk of confounding because matching was performed only on age, sex, and primary care practice, and groups remained unbalanced after matching. Moreover, groups were not matched based on medication class or other patient characteristics, and the analysis was not restricted to new users or to patients receiving only 2 antihypertensive medications at baseline, thus permitting important selection effects. Furthermore, adherence was not assessed in their study.

We addressed limitations in the existing literature and employed several methodologic approaches to improve the validity of comparisons in nonrandomized samples [34]. First, we used a similar active comparator. We compared FDC therapy with multipill combinations of the same medications, thus reducing potential confounding related to medication class. There was no compelling clinical reason to choose FDC or multipill combination therapy, and both are guideline-recommended, which suggests that there should be minimal indication bias. Second, we employed a new user study design and excluded patients with recent hospitalization or those who started combination medications on separate days to reduce the risk of selection bias. Third, we employed high-dimensional propensity score matching, which resulted in groups that were well-balanced on baseline characteristics. Finally, we used cataract surgery as a tracer outcome to assess for residual confounding and identified no difference between the groups on this measure. Therefore, we were able to rigorously examine the association between FDC use and clinical outcomes in a real-world setting.

The only residual differences in our propensity score–matched groups were in the index medications. These were due to differences between the commonly used medications in FDC and multipill formulations. Individuals in the FDC group were more likely to receive an ARB (65.1% versus 23.3% in the multipill combination group). There was a smaller difference in the use of hydrochlorothiazide (88.2% in FDC group versus 82.9% in multipill combination group). This is unlikely to explain the difference in primary outcome. Although ARBs have been associated with a lower incidence of adverse effects than ACEI, the 2 medication classes have similar effectiveness for treating hypertension with no consistent differences in clinical outcomes [35,36]. Although there is controversy about the comparative effectiveness of chlorthalidone and hydrochlorothiazide [19], the differences in thiazide use between the 2 groups in our study were small and would have biased outcomes in favor of the multipill group where chlorthalidone use was more common. Adjusting for the differences in index medications would be inappropriate because this would adjust away the effect of the broader exposure group. Importantly, outcomes were not different between the groups in the on-treatment analysis, which suggests that the observed differences were attributable to adherence and not to the medications themselves.

There are several limitations to our study. First, the observed difference in the occurrence of the primary composite endpoint was driven by fewer deaths in the FDC group. We did not observe significantly fewer occurrences of the individual cardiovascular events. This may be explained by the relatively low event rate (individual cardiovascular events occurred in 1.4% to 2.4% of participants) and limitations in sample size. Our study included only new users over the age of 66 years, and treatment for hypertension is often initiated earlier in life. By excluding individuals with prior hospitalizations for cardiovascular events and by restricting our sample to new users of antihypertensive medications, we likely selected for a lower-risk population, which may have affected our ability to identify clinically important differences in individual cardiovascular endpoints or safety outcomes. Second, we were unable to identify cause of death and thus could not distinguish between cardiovascular death and all-cause mortality. Although this limits our ability to explain the observed reductions in mortality, our findings are consistent with previous literature demonstrating that hypertension control reduces both all-cause mortality and cardiovascular mortality [37]. Third, we did not have blood pressure measurements, which impaired our ability to adjust for the severity of baseline hypertension. We attempted to address this issue by matching based on initial medication dose, and the groups were well-balanced in this regard. Fourth, our measures of adherence were based on medication dispensing and assumed that medications were taken as prescribed. These are not direct measures of medication adherence but are considered acceptable measures in secondary analysis of clinical and administrative datasets [38]. Finally, despite employing multiple methodological approaches to address confounding, the possibility of residual confounding remains a limitation in this observational analysis.

Approximately 675 million people globally require combination antihypertensive therapy, and this number may grow as new guidelines call for more intensive blood pressure control [6]. Up to 40% of patients with hypertension in high-income countries are treated with multipill regimens [9], and this number may be higher in low- and middle-income countries [39]. Internationally, half of hypertension societies do not recommend FDC treatment [7]. Our study suggests that FDC formulations are associated with better medication adherence and clinical outcomes. Using FDC rather than multipill therapy represents a simple and potentially low-cost intervention that could substantially reduce the global burden of morbidity and mortality related to hypertension.

## Supporting information

S1 Checklist STROBE and RECORD checklist for “Fixed-dose combination antihypertensive medications, adherence, and clinical outcomes: A population-based retrospective cohort study”. (DOCX)

S1 Table Dose categorization for antihypertensive medication. (DOCX)

S2 Table Balance in the year of study enrollment in the FDC and multipill groups. FDC, single-pill fixed-dose combination.
(DOCX)

S3 Table Baseline characteristics in study cohort before matching. (DOCX)

S4 Table Medications dispensed in the last 90 days of follow-up categorized by discontinuation status within each exposure group. (DOCX)

S5 Table Medication use among individuals treated with multipill or FDC antihypertensive regimens; medication discontinuation sensitivity analysis. FDC, single-pill fixed-dose combination.
(DOCX)

S6 Table Administrative diagnostic codes for components of the primary outcome, based on the International Statistical Classification of Diseases and Related Health Problems, 9th revision and 10th revision, Canada (ICD-9 and ICD-10-CA). (DOCX)

S1 Dataset Creation and Analysis Plan Dataset creation and data analysis plan for “Fixed-dose combination antihypertensive medications, adherence, and clinical outcomes: A population-based retrospective cohort study,” edited for clarity. (DOCX)

## Abbreviations

ACEI: angiotensin-converting enzyme inhibitor
AMI: acute myocardial infarction
ARB: angiotensin II-receptor blocker
FDC: single-pill fixed-dose combination
ICES: Institute for Clinical Evaluative Sciences
TIA: transient ischemic attack
