# Supplementary material for: Fixed-dose combination antihypertensive medications, adherence, and clinical outcomes: A population-based retrospective cohort study
Source: PLoS Med. 2018 Jun 11;15(6):e1002584. doi: 10.1371/journal.pmed.1002584 (PMC5995349; doi:10.1371/journal.pmed.1002584)
Supplement: S1 Table — (DOCX) [file pmed.1002584.s002.docx]

**S1 Table.** Dose Categorization for Anti-Hypertensive Medication

| **Drug Name** | **Low Dose (mg)** | **High Dose (mg)** |
| --- | --- | --- |
| Benazepril | <40 | ≥40 |
| Captopril | <100 | ≥100 |
| Cilazapril | <5 | ≥ 5 |
| Enalapril | <40 | ≥40 |
| Fosinopril | <40 | ≥40 |
| Lisinopril | <40 | ≥40 |
| Perindopril | <8 | ≥ 8 |
| Quinapril | <40 | ≥40 |
| Ramipril | <10 | ≥10 |
| Trandolapril | <8 | ≥8 |
| Candesartan | <32 | ≥32 |
| Eprosartan | <800 | ≥800 |
| Irbesartan | <300 | ≥300 |
| Losartan | <100 | ≥100 |
| Olmesartan | <40 | ≥40 |
| Telmisartan | <80 | ≥80 |
| Valsartan | <320 | ≥320 |
| Chlorthalidone | <25 | ≥25 |
| Hydrochlorothiazide | <25 | ≥25 |
| Indapamide | <2.5 | ≥2.5 |

**S1 Table Legend.** Each anti-hypertensive medication was categorized into high and low dose categories. High dose was defined as greater than or equal to the upper limit of the usual dose range as described in the American Society of Hypertension and the International Society of Hypertension Clinical Practice Guidelines for the Management of Hypertension in the Community.^1^ The one exception was hydrochlorothiazide, for which the upper limit of the usual dose range was described as 50 mg daily but we defined high dose as greater than or equal to 25 mg, because fewer than 5% of patients received a dose greater than 25 mg. A dose of 25 mg was used by approximately 20% of participants.

**Reference**

1. Weber MA, Schiffrin EL, White WB, et al. Clinical Practice Guidelines for the Management of Hypertension in the Community. J Clin Hypertens. 2014;16(1):14-26. doi:10.1111/jch.12237.
