# Supplementary material for: Fixed-dose combination antihypertensive medications, adherence, and clinical outcomes: A population-based retrospective cohort study
Source: PLoS Med. 2018 Jun 11;15(6):e1002584. doi: 10.1371/journal.pmed.1002584 (PMC5995349; doi:10.1371/journal.pmed.1002584)
Supplement: S2 Table — FDC, single-pill fixed-dose combination. (DOCX) [file pmed.1002584.s003.docx]

**S2 Table.** Balance in the year of study enrollment in the FDC and multi-pill groups.

| **Year of study enrollment** | **Multi-pill**  **(N=6,675)** | **FDC**  **(N=6,675)** | **Standardized Difference** |
| --- | --- | --- | --- |
| **2004** | 723 (10.8%) | 722 (10.8%) | 0 |
| **2005** | 806 (12.1%) | 824 (12.3%) | 0.01 |
| **2006** | 797 (11.9%) | 772 (11.6%) | 0.01 |
| **2007** | 733 (11.0%) | 688 (10.3%) | 0.02 |
| **2008** | 637 (9.5%) | 668 (10.0%) | 0.02 |
| **2009** | 612 (9.2%) | 595 (8.9%) | 0.01 |
| **2010** | 564 (8.4%) | 574 (8.6%) | 0.01 |
| **2011** | 522 (7.8%) | 534 (8.0%) | 0.01 |
| **2012** | 426 (6.4%) | 424 (6.4%) | 0 |
| **2013** | 455 (6.8%) | 450 (6.7%) | 0 |
| **2014** | 400 (6.0%) | 424 (6.4%) | 0.01 |
