# Supplementary material for: Fixed-dose combination antihypertensive medications, adherence, and clinical outcomes: A population-based retrospective cohort study
Source: PLoS Med. 2018 Jun 11;15(6):e1002584. doi: 10.1371/journal.pmed.1002584 (PMC5995349; doi:10.1371/journal.pmed.1002584)
Supplement: S3 Table — (DOCX) [file pmed.1002584.s004.docx]

**S3 Table.** Baseline characteristics in study cohort before matching.

| **Characteristic** | **Multi-Pill**  **(n=7,532)** | **FDC**  **(n=23,976)** | **Standardized Difference** |
| --- | --- | --- | --- |
| **Age, median (IQR), y** | 72 (68-78) | 71 (68-76) | 0.13 |
| **Female, n (%)** | 4,184 (55.5) | 12,872 (53.7) | 0.04 |
| **Neighborhood Income Quintile, n (%)** |  |  |  |
| 1 | 1,577 (20.9) | 4,694 (19.6) | 0.03 |
| 2 | 1,548 (20.6) | 5,025 (21.0) | 0.01 |
| 3 | 1,467 (19.5) | 4,730 (19.7) | 0.01 |
| 4 | 1,386 (18.4) | 4,776 (19.9) | 0.04 |
| 5 | 1,495 (19.8) | 4,589 (19.1) | 0.02 |
| Missing | 59 (0.8) | 162 (0.7) | 0.01 |
| **Nursing Home Residence (n, %)** | 267 (3.5) | 106 (0.4) | 0.22 |
| **Rural Residence, n (%)** | 1,109 (14.7) | 2,599 (10.8) | 0.12 |
| **Charlson comorbidity score, categorized** |  |  |  |
| No Hospitalizations | 6,266 (83.2) | 21,802 (90.9) | 0.23 |
| 0 | 670 (8.9) | 1,392 (5.8) | 0.12 |
| 1 | 302 (4.0) | 365 (1.5) | 0.15 |
| 2+ | 294 (3.9) | 417 (1.7) | 0.09 |
| **Healthcare Utilization** |  |  |  |
| Hospitalizations in prior year, mean (SD) | 0.16 (0.43) | 0.06 (0.27) | 0.27 |
| Outpatient physician visits in prior year, median (IQR) | 4 (2-8) | 5 (2-10) | 0.22 |
| Visit to cardiologist in prior 3 months, n (%) | 1,414 (18.8) | 4,067 (17.0) | 0.05 |
| Cardiac catheterization, in prior 5 years, n (%) | 85 (1.1) | 216 (0.9) | 0.02 |
| Total number of different prescription drugs in prior 100 days, mean (SD) | 1.50 (2.22) | 1.78 (2.26) | 0.12 |
| **Medical Comorbidities, n (%)** |  |  |  |
| Diabetes^†^ | 1,332 (17.7) | 4,277 (17.8) | 0 |
| Stroke^††^ | 81 (1.1) | 82 (0.3) | 0.09 |
| Acute myocardial infarction^††^ | 30 (0.4) | 47 (0.2) | 0.04 |
| Heart failure^††^ | 66 (0.9) | 83 (0.3) | 0.07 |
| Peripheral vascular disease^††^ | 57 (0.8) | 103 (0.4) | 0.04 |
| Chronic kidney disease^††^ | 31 (0.4) | 50 (0.2) | 0.04 |
| Cancer^†^ | 797 (10.6) | 2,666 (11.1) | 0.02 |
| Chronic obstructive pulmonary disease^†^ | 459 (6.1) | 1,390 (5.8) | 0.01 |
| Dementia^††^ | 609 (8.1) | 914 (3.8) | 0.18 |
| **Index Medication Use, n (%)** |  |  |  |
| ACEI | 5,868 (77.9) | 10,120 (42.2) | 0.78 |
| ARB | 1,664 (22.1) | 13,856 (57.8) | 0.78 |
| Hydrochlorothiazide | 6,309 (83.8) | 18,974 (79.1) | 0.12 |
| Chlorthalidone | 87 (1.2) | 0 (0.0) | 0.15 |
| Indapamide | 1,136 (15.1) | 5,002 (20.9) | 0.15 |
| **Index Medication Dose Category, n (%)** |  |  |  |
| Low | 2,987 (39.7) | 16,650 (69.4) | 0.63 |
| Medium | 3,445 (45.7) | 4,930 (20.6) | 0.56 |
| High | 1,100 (14.6) | 2,396 (10.0) | 0.14 |
| **Other Medications in prior 100 days, n (%)** |  |  |  |
| Non-insulin antihyperglycemic | 812 (10.8) | 2,274 (9.5) | 0.04 |
| Insulin | 110 (1.5) | 258 (1.1) | 0.03 |
| Statin | 1,939 (25.7) | 6,453 (26.9) | 0.03 |
| Warfarin | 170 (2.3) | 277 (1.2) | 0.09 |
| Direct Oral Anticoagulants | 11 (0.1) | 41 (0.2) | 0.01 |
| Digoxin | 50 (0.7) | 100 (0.4) | 0.03 |
| Clopidogrel | 91 (1.2) | 188 (0.8) | 0.04 |
