# Supplementary material for: Fixed-dose combination antihypertensive medications, adherence, and clinical outcomes: A population-based retrospective cohort study
Source: PLoS Med. 2018 Jun 11;15(6):e1002584. doi: 10.1371/journal.pmed.1002584 (PMC5995349; doi:10.1371/journal.pmed.1002584)
Supplement: S4 Table — (DOCX) [file pmed.1002584.s005.docx]

**S4 Table.** Medications dispensed in the last 90 days of follow-up categorized by discontinuation status within each exposure group.

|  | **FCG** | | **FDC** | |
| --- | --- | --- | --- | --- |
| **Medication Class** | **Discontinued*** | **Did not discontinue** | **Discontinued*** | **Did not discontinue** |
|  | **N=s** | **N=754** | **N=5,544** | **N=1,131** |
| Index antihypertensive | 1,349 (22.8%) | 648 (85.9%) | 1,759 (31.7%) | 997 (88.2%) |
| Other antihypertensive | 1,783 (30.1%) | 142 (18.8%) | 1,669 (30.1%) | 239 (21.1%) |
| Non-insulin | 708 (12.0%) | 105 (13.9%) | 739 (13.3%) | 151 (13.4%) |
| Insulin | 134 (2.3%) | 15 (2.0%) | 145 (2.6%) | 25 (2.2%) |
| Statin | 1,892 (32.0%) | 317 (42.0%) | 1,717 (31.0%) | 431 (38.1%) |
| Warfarin | 209 (3.5%) | 22 (2.9%) | 177 (3.2%) | 33 (2.9%) |
| Direct Oral Anticoagulant | 78 (1.3%) | 8 (1.1%) | 70 (1.3%) | 15 (1.3%) |
| Digoxin | 66-71** | ≤5** | 51 (0.9%) | 12 (1.1%) |
| Clopidogrel | 193 (3.3%) | 22 (2.9%) | 180 (3.2%) | 21 (1.9%) |

S4 Table Legend: Medication discontinuation was defined as having any break of greater than 150% of the previous days’ supply. *Values censored to comply with ICES privacy policies because calculations required cells with 5 or fewer observations.
