# Supplementary material for: Fixed-dose combination antihypertensive medications, adherence, and clinical outcomes: A population-based retrospective cohort study
Source: PLoS Med. 2018 Jun 11;15(6):e1002584. doi: 10.1371/journal.pmed.1002584 (PMC5995349; doi:10.1371/journal.pmed.1002584)
Supplement: S5 Table — FDC, single-pill fixed-dose combination. (DOCX) [file pmed.1002584.s006.docx]

**S5 Table.** Medication use among individuals treated with multi-pill or FDC antihypertensive regimens, medication discontinuation sensitivity analysis

|  | **Multi-pill** | **FDC** |
| --- | --- | --- |
| **Medication Use** | **N=6,675** | **N=6,675** |
| **Follow-up time, median (IQR), days** | 1826 (1142-1826) | 1826 (1163-1826) |
| **Time to initial discontinuation, median (IQR), days*** | 280 (73-1285) | 556 (125-**)† |
| **Proportion of days covered in all periods of continuous use, median (IQR)** | 0.45 (0.11-0.99) | 0.75 (0.20-1.00)^†^ |

S5 Table Legend: Time to initial discontinuation represents the first period of continuous medication use, defined as no disruption of greater than 300% of the previous days’ supply in receiving the index medications. *Indicates Kaplan-Meier estimate. **Third quartile cannot be estimated as > 25% were still on therapy at that time. IQR: Inter-quartile range, FDC: Single-pill Fixed-Dose Combination. †Indicates p<0.01 for between-group difference.
