# Supplementary material for: Fixed-dose combination antihypertensive medications, adherence, and clinical outcomes: A population-based retrospective cohort study
Source: PLoS Med. 2018 Jun 11;15(6):e1002584. doi: 10.1371/journal.pmed.1002584 (PMC5995349; doi:10.1371/journal.pmed.1002584)
Supplement: S6 Table — (DOCX) [file pmed.1002584.s007.docx]

**S6 Table.** Administrative diagnostic codes for components of the primary outcome, based on the International Statistical Classification of Diseases and Related Health. Problems, Ninth Revision and Tenth Revision, Canada (ICD-9 and ICD-10-CA).

| Outcome | Administrative codes |
| --- | --- |
| Stroke | ICD-9: 430, 431, 434, 3623  ICD-10-CA: H34.1, I60.x (excl 160.8), I61.x, I63.x, I64.x |
| Acute myocardial infarction | ICD-9: 410x  ICD-10-CA: I21.x, I22.x |
| Heart failure | ICD-9: 428x  ICD-10-CA: I09.9, I25.5, I42.0, I42.5 – I42.9, I43.x, I50.x, P29.0 |
| Death | All cause death from Ontario Registered Persons Database. |
