# Supplementary material for: Fixed-dose combination antihypertensive medications, adherence, and clinical outcomes: A population-based retrospective cohort study
Source: PLoS Med. 2018 Jun 11;15(6):e1002584. doi: 10.1371/journal.pmed.1002584 (PMC5995349; doi:10.1371/journal.pmed.1002584)
Supplement: S1 Dataset Creation and Analysis Plan — (DOCX) [file pmed.1002584.s008.docx]

**S1 Dataset Creation and Analysis Plan.** Dataset creation and data analysis plan for “Fixed-dose combination antihypertensive medications, adherence, and clinical outcomes: A population-based retrospective cohort study”, edited for clarity.

| Project InitiationThis Section must be Completed Prior to Project Dataset(s) Creation | | | | | |
| --- | --- | --- | --- | --- | --- |
| **Project Title:** | The Use and Effectiveness of Fixed-Dose Combination Antihypertensive Medications | | | | |
| **Project TRIM number:** | 2017 0760 243 000 | | | | |
| **Research Program:** | CDP | | | | |
| **Site:** | ICES Central | | | | |
| **Project Objectives:** | *Insert Project Objectives as listed in the approved ICES Project PIA* | | | | |
|  | 1. To examine utilization and persistent use of fixed-dose combination (FDC) antihypertensive medication. 2. To examine the effectiveness of FDC antihypertensive medication compared with free combinations of antihypertensives in reducing hypertension-related complications. | | | | |
| **ICES Project PIA Initial Approval Date:** | *The ICES Employee or agent who is responsible for creating the Project Dataset(s) is responsible for ensuring there is an approved ICES Project PIA and verifying the date of approval prior to creating the Project Dataset(s)* | | | | |
|  | 2016-SEP-22 | | | | |
| **Principal Investigator (PI):** | Amol Verma | | | | |
| **Check the applicable box if the PI is an ICES Student/Trainee** | ICES Student  ICES Fellow  ICES Post-Doctoral Trainee  Visiting Scholar | | | | |
| **Responsible ICES Scientist:** | *Name the Responsible ICES Scientist if the PI is not a Full Status ICES Scientist* | | | | |
|  | Muhammad Mamdani | | | | |
| **Project Team Member(s) Responsible for Project Dataset Creation and/or Statistical Analysis and date joined (list all):** | *All person(s) (ICES Analyst, Appointed Analyst, Analytic Epidemiologist, PI, and/or Student) responsible for creating the Project Dataset(s) and/or statistical analysis on the Research Analytics Environment (RAE) and the date they joined the project must be recorded* | | | | |
|  | Tara Gomes, Wayne Khuu | | | 2016-AUG-22 | |
| **Other ICES Project Team Members and date joined (list all):** | *All other Research Project Team Members (e.g., Research Administrative Assistants, Research Assistants, Project Managers, Epidemiologists) and the date they joined the project must be recorded* | | | | |
|  | Mina Tadrous, Samantha Singh | | | 2016-AUG-22 | |
| **Confirmation that DCP is consistent with Project Objectives:** | *The following individuals must confirm that the ICES Data provided for in this DCP is relevant (e.g., with respect to cohort, timeframe, and variables) and required to achieve the Project Objectives stated in the ICES Project PIA prior to initial Project Dataset creation: 1) PI; 2) Responsible ICES Scientist if the PI is not a Full Status ICES Scientist, or a second ICES Scientist or the Scientific Program Lead if the PI is creating both the DCP and the Project Dataset[s]; 3) ICES Research and Analysis Staff creating the DCP; and 4) ICES Analytic Staff (ICES Employee or agent responsible for creating the Project Dataset[s]). This may be delegated either verbally or via e-mail.* | | | | |
|  | ***Principal Investigator*** | |  | | 2016-Nov-21 |
|  | ***Responsible ICES Scientist or Second ICES Scientist/Lead*** | |  | 2016-Nov-21 | |
|  | ***ICES Research and Analysis Staff Creating the DCP*** | |  | 2017-JAN-13 | |
|  | ***ICES Analytic Staff*** | |  | 2017-JAN-13 | |
| **Designated ICES Research and Analysis Staff accountable for Project Documentation:** | *The person named (ICES staff) is accountable for ensuring that the approved ICES Project PIA, ICES Project PIA Amendments, and DCP are saved on the T Drive, ensuring ICES Project PIA Amendments are submitted as required, ensuring DCP Amendments are documented, and sharing the final DCP with the PI/Responsible ICES Scientist at project completion* | | | | |
|  | Wayne Khuu | | | | |
| **DCP Creation Date and Author:** | *Date DCP was finalized prior to Project Dataset(s) creation* | *Name of person who created the DCP* | | | |
|  | ***Date*** | ***Name*** | | | |
|  | 2016-AUG-22 | Amol Verma | | | |

| ICES DataThis Section must be Completed Prior to Project Dataset(s) Creation | |
| --- | --- |
| *The ICES Employee or agent who is responsible for creating the Project Dataset(s) must ensure that this list includes only data listed in the ICES Project PIA*  *Changes to this list after initial ICES Project PIA approval require an ICES Project PIA Amendment* | *Mandatory for all datasets that are available by individual year* |
| ***General Use Datasets – Health Services*** | ***Years (where applicable)*** |
| ODB | 2003-2015 |
| CIHI DAD | 2003-2015 |
| NACRS | 2003-2015 |
| CIHI SDS | 2003-2015 |
| OHIP | 2003-2015 |
|  |  |
| ***General Use Datasets – Care Providers*** |  |
| IPDB | 2003-2015 |
| ***General Use Datasets – Population*** |  |
| RPDB | 2003-2015 |
| See list |  |
| ***General Use Datasets – Coding/Geography*** |  |
| DIN |  |
| REF |  |
| PCCF |  |
| ***General Use Datasets - Facilities*** |  |
| See list |  |
| ***General Use Datasets - Other*** |  |
| CHF |  |
| COPD |  |
| ODD |  |
| OMID |  |
| CHF |  |
| ***Controlled Use Datasets*** |  |
| OCR |  |
| See list |  |
| ***Other Datasets*** |  |
|  |  |

| Project Cohort | | |
| --- | --- | --- |
| **Study Design** | Cohort study  Matched cohort study  Case-control study  Cross-sectional study  Other (specify): | |
| **Index Event / Inclusion Criteria** | Index event: First prescription of fixed-dose combination (FDC) (Dclassnew = 1,2,3,4) or free combination of anti-hypertensive medications.   - Mutually exclusive groups   Index date = date of first prescription for FDC or free combination medication  Note: For accrual, get all ODB records for cohort drugs where age>=65 on servdate and valikn=‘V’  Definitions:  FDC medication: Combination ACE-inhibitor or ARB + Thiazide (See medication list attached; DCLASSNEW = 1,2,3,or 4)  Free combination group: New users of free combination of anti-hypertensive medications. The combination must be either ACE-I or ARB plus a Thiazide (see medication list). Both medications must have been started on the same day. ACE-I = DCLASSNEW 5, ARB = DCLASSNEW 6, Thiazide = DCLASSNEW 7,8,9, or 10. Therefore, eligible users are (DCLASSNEW 5 or 6) AND (DCLASSNEW 7,8,9, or 10). | |
| **Estimated Size of Cohort**  **(if known)** | Based on feasibility analysis, the expected cohort size is:   - FDC Group: 28,694 - Free Combination Group: 12,897 (ACE-I + Thiazide 10,114; ARB + Thiazide 2,783)   Note that the sample size for the feasibility analysis did not exclude individuals based on criteria 4-6 below and did not include any matching between the groups, so study sample will be smaller. | |
| **Exclusions (in order)** | *Step* | Description |
|  | 1 | Age < 66 years |
|  | 2 | No valid IKN: valikn not=V (Performed at record level when extracting ODB) |
|  | 3 | Individuals with a prescription for any anti-hypertensive medication in the 12 months prior to index date. (DClassNew 1-11) |
|  | 4 | Individuals prescribed any additional anti-hypertensive medication other than the FDC or free combination on the index date (DCLASSNew 1-11)  Note: This was split into 3 steps, see result file for details |
|  | 5 | Individuals with hospitalization where Most Responsible Diagnosis was stroke or TIA, heart failure, or myocardial infarction in the 1 year prior to index date, including index date. (ICD 10-CA Codes in Appendix) |
|  | 6 | Individuals with Emergency Department visit where Most Responsible Diagnosis is stroke or TIA in the 1 year prior to index date, including index date. |
|  | 7 | Death on or prior to index date |

| Project Time Frame Definitions | | |
| --- | --- | --- |
| Look-back Window  Observation Window  (in which to look for outcomes)  **Index Event Date**  Accrual Window  Max Follow-up Date | |  |
| **Accrual Start/End Dates** | April 1, 2004 – December 31, 2014 |  |
| **Max Follow-up Date** | March 31^st^, 2015 |  |
| **When does observation window terminate?** | 5 years after index date or March 31^st^, 2015 |  |
| **Lookback Window(s)** | 1 year for exclusions  100 days to 5 years for baseline characteristics |  |

| Variable Definitions (add additional rows as needed) | | | |
| --- | --- | --- | --- |
| **Main Exposure or Risk Factor** | | Use of FDC vs. free dose combination antihypertensive medications (see definition above.  Define ongoing use of FDC and free dose combinations as:   - Discontinuation of anti-hypertensive medication: No repeat prescription within 150% of previous days supply. For free combination medications, no repeat prescription for either one or both drugs within the 150% grace period is considered discontinuation. - Allow switching between ACEI/ARB - Define end date for FDC group as: last prescription date + 150% of days supply of last prescription. - Define end date for Free Combination group as: last prescription date of the first discontinued medication + 150% of days supply of the last prescription of the discontinued medication. |  |
| **Propensity Score Matching** | Generate a High-Dimensional Propensity Model using the following dimensions:   - - 1 year ODB claims (based on drug name – *EXCLUDE anti-hypertensive medications: DCLASS = 1-11*)   - 1 year CIHI-DAD data (dxcode and incode – ONLY KEEP 5 DIGITS OF CCI CODES AND 3 DIGITS OF DXCODES) [Consider dxcodes and incodes as 2 separate dimensions]     1. Note: 3 Digits = length3= [e.g.,] S01. NOT S012     2. Source=Inpatient     3. Acute=T   - 1 year CIHI-NACRS data (dxcode and incode – ONLY KEEP 5 DIGITS OF CCI CODES AND 3 DIGITS OF DXCODES) [consider dxcodes and incodes as 2 separate dimensions]     1. Source=ED,     2. Include suspect=T     3. Include scheduled=T   - OHIP     1. Source=all     2. Spec=Medical     3. 1 year OHIP feecodes     4. 1 year OHIP dxcodes   - The 200 most prevalent empirical covariates to consider from each of the above datasets   - The number of empirical covariates to include in the resulting propensity score = 500 - FORCE INTO MODEL:   - Demographic variables: age, sex, Income Quintile, Year of index date   - Charlson Comorbidity Index (categories)   - Number of outpatient physician visits in 1 year prior to index date     1. OHIP claims where location= ‘O’     2. Source=nonlab     3. Spec=physician     4. Keep 1 record per patient per physnum per day   - At least one cardiology visit in 3 months prior to index date. **NOTE: Wayne, please check the proportion of study participants who had a cardiology visit. If too low, we may need to extend the lookback window.     1. 3 month lookback is OK - ***NOTE: THE COHORT INPUT INTO THE HDPS MACRO CAN ONLY INCLUDE VARIABLES TO BE USED IN THE PROPENSITY SCORE ANALYSIS, EXPOSURE VARIABLE, AND OUTCOME VARIABLE. OTHERWISE, MACRO WILL NOT WORK PROPERLY. Use the primary outcome as defined below in the macro.   **Matching Variables:**  Matching Ratio: Match 1: 1 (FDC:Free Combinations)   - LOGIT HD Propensity score (within 0.2*STD(logit(hdps)) caliper) - Dose of Index ACEI/ARB AND thiazide (description on page 9) | |  |
| **Analysis Plan** | **‘Intention-to-treat’ analysis.**   - Time­to­event analyses using Cox proportional hazard models - Censoring criteria:   - Death (when not an outcome)   - End of follow-up period (5 years maximum)   - End of study period (March 31, 2015)   - Do not censor participants for drug discontinuation. - Primary and secondary outcomes for the main analysis are defined below - Matched analysis accounting for matched nature of data using a robust variance estimator that accounts for clustering within matched sets (Lin & Wei, 1989, cited in Austin 2013 DOI 10.1002/sim.5984)   ‘**On-Treatment’ analysis.**   - Same as main analysis, but also include discontinuation of anti-hypertensive medication as censoring criterion.   - Discontinuation defined as the first break in therapy (using 1.5x dayssupl lookforward)   **Post-hoc analysis to address reviewer comments from PLOS Medicine:**   - Cox regression for matched cohort, on-treatment analysis for primary, composite outcome   - Use 3x dayssupl grace period for continuous use instead of 1.5x   - Add 1.5x dayssupl to last prescription in period of continuous use to define end-date   - Calculate PDC estimates using 3x grace period definition for continuous use - Produce survival curve with number at risk for primary outcome in matched cohort, ITT analysis | |  |
| **Primary Outcome Definition** | | Composite endpoint of hospitalization for stroke, heart failure (CHF), acute myocardial infarction (AMI), or death. (ICD Codes in Appendix) |  |
| **Secondary Outcome Definition(s)** | | - Hospitalization for Stroke - Hospitalization for Heart Failure - Hospitalization for Myocardial infarction - Death (any cause from RPDB) - Safety Outcomes:   - Hospitalization for hypokalemia   - Hospitalization for hyponatremia - Tracer outcome: Cataract surgery - Discontinuation of anti-hypertensive medication: as defined above. In this analysis instead of censoring on discontinuation, it is the outcome of interest.   **For response to reviewers comments from PLOS Medicine:**   - Calculate rate of outcomes per 100,000 person-years follow-up as:   - [Sum of number of outcomes of interest across all persons / sum of days of follow up across all persons]*100000   - Stratified by exposure - Follow-up days calculated separately for each outcome as the number of days between index and end of follow-up, where follow-up calculated as:   - First date of: Death, Outcome of interest, End of study period (31 MAR2015), Max 5 year followup     - Additionally, for on-treatment analysis, date of first discontinuation of index therapy (no refill 1.5x dayssupl of previous prescription) |  |
| **Baseline Characteristics** | | - Age at index date - Gender - Neighborhood income quintile   - Missing, 1-5 - Living in long-term care home (based on LTC flag on index prescription) - Rural residence - Charlson Comorbidity   - CIHI-DAD (inpatient, acute care)   - Include MRDx in calculation of Charlson score   - Based on 2-year lookback, including index date   - Categorized as no hospitalization, 0, 1, 2, or 3+ - Cardiac Revascularization procedures – see appendix for codes   - 5-years lookback   - cardiac catheterization - Specific diseases (including index date use diagnosis date)   - ICES validated disease databases, diagdate<=indexdate     - DM - use ODD     - Malignancy - Diagnosis of cancer using Ontario Cancer Registry     - COPD – use COPD database       - Use specific COPD database   - 5 year look-back, including index date:     - Stroke (Note: no hospitalizations for this in 1 year prior to index date as per exclusion criteria) – use ICD codes in appendix     - TIA – codes in appendix     - Acute Myocardial Infarction (Note: no hospitalizations for this in 1 year prior to index date as per exclusion criteria)     - Heart Failure (Note:no hospitalizations for this in 1 year prior to index date as per exclusion criteria) – use CHF database     - Peripheral Vascular Disease – use codes in appendix     - Chronic Kidney Disease – use codes in appendix     - Dementia – use codes in appendix - Dose of ACE-inhibitor or ARB at index date. - Dose of Thiazide at Index Date   - Calculate daily dose = (quantity*strength)/dayssupl   - Daily dose of thiazide and ACEI or ARB separately, grouped by generic drug name.     - If two medications of same primary drug (e.g., BENAZEPRIL CHLOROHYDRATE with BENAZEPRIL HCL), then sum daily dose   - Tablulate cutoffs for tertiles by generic drug name   - Tablulate quantiles by 5 by generic drug name   - Amol classified dose into high/low based on the top of the 'usual dose range' as recommended by 2 hypertension guidelines and set this as "high" and everything else as "low" (see appendix) - Dose of index ACEI/ARB **and** thiazide   - Low = low dose of ACEI/ARB and thiazide   - Medium = Low dose of one, high dose of the other   - High = high dose of ACEI/ARB and thiazide - Index use of anti-hypertensive medications:   - ACE-inhibitor (DCLASSNEW = 1,2, or 5)   - ARB (DCLASSNEW = 3,4,6)   - Hydrochlorothiazide (DCLASSNEW = 1,3,9)   - Chlorthalidone (DCLASSNEW = 4,8)   - Indapamide (DCLASSNEW = 2,10) - Using other medications (medications dispensed in 100 day lookback including index date)   - Non-insulin oral antihyperglycemic agents (metformin, sulfonylureas, thiazoladinediones, meglitinides, acarbose, DPP4-inhibitors)   - Insulin   - Statin   - Warfarin   - Novel Oral Anticoagulant   - Loop Diuretic   - Digoxin   - Clopidogrel - Total number of distinct medications dispensed in 100 day lookback NOT including index date (based on drug name)   - Median, IQR per participant - Healthcare Utilization   - Number of hospitalizations in year prior to index date (CIHI-DAD Inpatient, acute care)     - Count number of distinct episodes of care   - Number of outpatient physician visits in year prior to index date (OHIP, outpatient defined using OHIP_LOCATION macro - OFFICE ONLY; source=nonlab, spec=physician)     - Count one record per patient per physician per day     - Use OHIP physnum, link to IPDB, use mainspec=’CARDIOLOGY’     - Location = “O”     - Count 1 record per patient per physician per day   - At least one cardiology visit in 3 months prior to index date (yes/no) |  |
| **Other Variables** | | - For both matched and unmatched cohorts, main Intention-to-treat analysis only: Proportion of days covered.   - Calculate for the first period of continuous use as well as all periods of continuous use.   - Defined as: Days supply* of FDC or number of days supply when BOTH Free-Combination medications were prescribed divided by the follow up time:     - *Note: Days supply is the days within the period of continuous use, including the grace period days, not adding left over days supply for early refils   - Sum of days supply / days of follow-up     - Days follow up calculated as would be the number of days between index date and the first of: death, reaching the end of study period (31MAR2015), maximum follow-up (5 years).   - Sum of days of follow up (matched cohort only)   - For PDC, calculate p-values for matched cohort using Wilcoxon signed rank sum test. - For main Intention-to-treat analysis only: Median time to discontinuation of continuous use, as defined above, using Kaplan Meier estimates.   **Post-hoc variables to address PLOS Medicine reviewer comments:**   - N(%) who received other BP modifying medications in the last 90 days of follow-up   1. End of follow-up calculated for each person as first of: death date, end of study period (31MAR2015), Maximum 5 years follow-up from index   2. Medications:      1. Index medication:         1. For FDC: DCLASS=1 or DCLASS=2         2. For FCG: (DCLASS=3 or DCLASS=4 and DCLASS=5      2. Other anti-hypertensives (dclass=6)      3. Non-insulin (dclass=NONINSULIN)      4. Insulin (dclass=INSULIN)      5. Statins (dclass=STATIN)      6. Warfarin (dclass=WARFARIN)      7. DOAC (dclass=DOAC)      8. Digoxin (dclass=Digoxin)      9. Clopidogrel (dclass=CLOPIDOGREL) - Stratification: - 1. By exposure and calculate standardized difference - 2. By discontinuation status (i.e., had a break in therapy > 1.5x dayssupl) within exposure group |  |
|  | |  |  |

| Analysis Plan and Dummy Tables (expand/modify as needed) | |
| --- | --- |
| **Descriptive Tables (insert or append dummy tables), e.g.:** | |
| **Dummy tables below** | |
| **Statistical Model(s)** | |
| **Type of model** | Time­to­event analyses using Cox proportional hazard models accounting for matched nature of data |
| **Primary independent variable** | Anti-hypertensive group (FDC vs. free combination) |
| **Dependent variable** | Composite primary endpoint as described above |
| **Sensitivity Analyses** | ‘On treatment analysis’ |
| **Type of model** | Same as main analysis, but include discontinuation of anti-hypertensive medication as censoring criterion. |
| **Primary independent variable** | Anti-hypertensive group (FDC vs. free combination) |
| **Dependent variable** | Composite primary endpoint as described above |
|  | |

**Table 1a. Baseline characteristics before matching.**

| **Variable** | **FDC** | **Free Combination** | **Standardized Difference** |
| --- | --- | --- | --- |
|  | N= | N= |  |
| Age at cohort entry (Median, IQR) |  |  |  |
| Gender (Female) | N (%) | N (%) |  |
| Neighborhood income quintile |  |  |  |
| 1 | N (%) | N (%) |  |
| 2 | N (%) | N (%) |  |
| 3 | N (%) | N (%) |  |
| 4 | N (%) | N (%) |  |
| 5 | N (%) | N (%) |  |
| Living in Long-Term Care | N (%) | N (%) |  |
| Rural Residence | N (%) | N (%) |  |
| Comorbidity (Romano-Charlson or Deyo-Charlson Score) |  |  |  |
| No hospitalization |  |  |  |
| 0 | N (%) | N (%) |  |
| 1 | N (%) | N (%) |  |
| 2+ | N (%) | N (%) |  |
| Cardiac Revascularization procedures |  |  |  |
| cardiac catheterization | N (%) | N (%) |  |
| Other Comorbidities |  |  |  |
| DM | N (%) | N (%) |  |
| Stroke | N (%) | N (%) |  |
| MI | N (%) | N (%) |  |
| Heart Failure | N (%) | N (%) |  |
| Peripheral Vascular Disease | N (%) | N (%) |  |
| Chronic Kidney Disease | N (%) | N (%) |  |
| Malignancy | N (%) | N (%) |  |
| COPD | N (%) | N (%) |  |
| Dementia | N (%) | N (%) |  |
| Dose of anti-hypertensive drugs |  |  |  |
| Low | N (%) | N (%) |  |
| Medium | N (%) | N (%) |  |
| High | N (%) | N (%) |  |
| Index anti-hypertensive medications used: |  |  |  |
| ACE-inhibitor | N (%) | N (%) |  |
| ARB | N (%) | N (%) |  |
| Hydrochlorothiazide | N (%) | N (%) |  |
| Chlorthalidone | N (%) | N (%) |  |
| Indapamide | N (%) | N (%) |  |
| Other medications used in past 100 days |  |  |  |
| Non-insulin oral antihyperglycemics (any of metformin, sulfonylureas, thiazoladinediones, meglitinides, acarbose, DPP4-inhibitors) | N (%) | N (%) |  |
| Insulin | N (%) | N (%) |  |
| Statin | N (%) | N (%) |  |
| Warfarin | N (%) | N (%) |  |
| Novel Oral Anticoagulant | N (%) | N (%) |  |
| Loop Diuretic | N (%) | N (%) |  |
| Digoxin | N (%) | N (%) |  |
| Clopidogrel | N (%) | N (%) |  |
| Number of medications in past 100 days | Median (IQR) | Median (IQR) |  |
| Number of hospitalizations in 1 years prior to index date | Median (IQR) | Median (IQR) |  |
| Number of outpatient physician visits in 1 year prior to index date | Median (IQR) | Median (IQR) |  |
| At least one cardiology visit in 3 months prior to index date (yes/no) | N(%) | N(%) |  |

**Table 1b. Baseline characteristics after propensity score matching.**

| **Variable** | **FDC** | **Free Combination** | **Standardized Difference** |
| --- | --- | --- | --- |
|  | N= | N= |  |
| Age at cohort entry (Median, IQR) |  |  |  |
| Gender (Female) | N (%) | N (%) |  |
| Neighborhood income quintile |  |  |  |
| 1 | N (%) | N (%) |  |
| 2 | N (%) | N (%) |  |
| 3 | N (%) | N (%) |  |
| 4 | N (%) | N (%) |  |
| 5 | N (%) | N (%) |  |
| Living in Long-Term Care | N (%) | N (%) |  |
| Rural Residence | N (%) | N (%) |  |
| Comorbidity (Romano-Charlson or Deyo-Charlson Score) |  |  |  |
| No hospitalization |  |  |  |
| 0 | N (%) | N (%) |  |
| 1 | N (%) | N (%) |  |
| 2+ | N (%) | N (%) |  |
| Cardiac Revascularization procedures |  |  |  |
| cardiac catheterization | N (%) | N (%) |  |
| Other Comorbidities |  |  |  |
| DM | N (%) | N (%) |  |
| Stroke | N (%) | N (%) |  |
| MI | N (%) | N (%) |  |
| Heart Failure | N (%) | N (%) |  |
| Peripheral Vascular Disease | N (%) | N (%) |  |
| Chronic Kidney Disease | N (%) | N (%) |  |
| Malignancy | N (%) | N (%) |  |
| COPD | N (%) | N (%) |  |
| Dementia | N (%) | N (%) |  |
| Dose of anti-hypertensive drugs |  |  |  |
| Low | N (%) | N (%) |  |
| Medium | N (%) | N (%) |  |
| High | N (%) | N (%) |  |
| Index anti-hypertensive medications used: |  |  |  |
| ACE-inhibitor | N (%) | N (%) |  |
| ARB | N (%) | N (%) |  |
| Hydrochlorothiazide | N (%) | N (%) |  |
| Chlorthalidone | N (%) | N (%) |  |
| Indapamide | N (%) | N (%) |  |
| Other medications used at index |  |  |  |
| Non-insulin oral antihyperglycemics (any of metformin, sulfonylureas, thiazoladinediones, meglitinides, acarbose, DPP4-inhibitors) | N (%) | N (%) |  |
| Insulin | N (%) | N (%) |  |
| Statin | N (%) | N (%) |  |
| Warfarin | N (%) | N (%) |  |
| Novel Oral Anticoagulant | N (%) | N (%) |  |
| Loop Diuretic | N (%) | N (%) |  |
| Digoxin | N (%) | N (%) |  |
| Clopidogrel | N (%) | N (%) |  |
| Number of medications in past 100 days | Median (IQR) | Median (IQR) |  |
| Number of hospitalizations in 1 years prior to index date | Median (IQR) | Median (IQR) |  |
| Number of outpatient physician visits in 1 year prior to index date | Median (IQR) | Median (IQR) |  |
| At least one cardiology visit in 3 months prior to index date (yes/no) | N(%) | N(%) |  |

**Table 2.** Follow-up and anti-hypertensive medication use in the main intention-to-treat analysis

|  | **FDC** | **Free Combination** |
| --- | --- | --- |
| Follow-up time | Median (IQR) | Median (IQR) |
| Time to discontinuation | Median | Median |
| Proportion of days covered | Median (IQR) | Median (IQR) |

**Table 3.** Primary and Secondary Outcomes in the intention-to-treat analysis

| **Outcome** | **N (%)**  **FDC** | **N (%)**  **Free Combination** | **Unadjusted** | | | **Adjusted** | | |
| --- | --- | --- | --- | --- | --- | --- | --- | --- |
|  |  |  | **HR** | **Lower CL** | **Upper CL** | **HR** | **Lower CL** | **Upper CL** |
| **Primary Outcome:**  Composite of AMI, CHF, Stroke or Death |  |  |  |  |  |  |  |  |
| **Secondary Outcomes:** |  |  |  |  |  |  |  |  |
| AMI |  |  |  |  |  |  |  |  |
| CHF |  |  |  |  |  |  |  |  |
| Stroke |  |  |  |  |  |  |  |  |
| Death |  |  |  |  |  |  |  |  |
| Drug Discontinuation |  |  |  |  |  |  |  |  |
| **Safety Outcomes:** |  |  |  |  |  |  |  |  |
| Hypokalemia |  |  |  |  |  |  |  |  |
| Hyponatremia |  |  |  |  |  |  |  |  |
| **Tracer Outcome:**  Cataract Surgery |  |  |  |  |  |  |  |  |

**Figure 1a.** Kaplan-Meier curve for drug discontinuation in FDC and Free Combination groups.

**Figure 1b.** Kaplan-Meier curve for primary outcome in FDC and Free Combination groups in intention-to-treat analysis.

**Supplementary Material:**

**sTable 1.** Primary and Secondary Outcomes in the On-treatment analysis

| **Outcome** | **N**  **FDC** | **N**  **Free Combination** | **Unadjusted** | | | **Adjusted** | | |
| --- | --- | --- | --- | --- | --- | --- | --- | --- |
|  |  |  | **HR** | **Lower CL** | **Upper CL** | **HR** | **Lower CL** | **Upper CL** |
| **Primary Outcome:**  Composite of AMI, CHF, Stroke or Death |  |  |  |  |  |  |  |  |
| **Secondary Outcomes:** |  |  |  |  |  |  |  |  |
| AMI |  |  |  |  |  |  |  |  |
| CHF |  |  |  |  |  |  |  |  |
| Stroke |  |  |  |  |  |  |  |  |
| Death |  |  |  |  |  |  |  |  |
| Drug Discontinuation |  |  |  |  |  |  |  |  |

| Quality Assurance Activities | | | | | |
| --- | --- | --- | --- | --- | --- |
| **RAE Directory of SAS Programs** | | /users/wkhuu/odprn/p0760.243.000/fullstudy | | | |
| **RAE Directory of Final Dataset(s)** | | *The* *final analytic dataset for each cohort includes all the data required to create the baseline tables and run all the models. It should include all covariates for all models such as patient risk factors, hospital characteristics, physician characteristics, exposure measures (continuous, categorical) and outcomes. It should include covariates that were considered but didn’t make the final cut. This would permit an analyst to easily re-run the models in the future.* | | | |
|  |  | /sasroot/projects/cdp/p0760.243.000/fullstudy | | | |
| **RAE README file available:** Yes No | | | | | |
| **Date results of quality assurance tools for final dataset shared with project team (where applicable):** | | | | |  |
| **%assign** | 2017-Feb-02 | |  |  |  |
| **%evolution** | yyyy-mon-dd | |  |  |  |
| **%dinexplore** | 2016-Sep-27 | |  |  |  |
| **%track / %exclude** | 2017-Feb-02 | |  |  |  |
| **%codebook** | 2017-Feb-02 | |  |  |  |
| **Additional comments:** | | | |  | |
